# Retracted: Clinical Application of Perioperative Anaesthesia Management Based on Enhanced Recovery after Surgery Concept to Elderly Patients Undergoing Total Knee Replacement

**DOI:** 10.1155/2023/9763172

**Published:** 2023-03-12

**Authors:** Computational Intelligence and Neuroscience

*Computational Intelligence and Neuroscience* has retracted the article titled “Clinical Application of Perioperative Anaesthesia Management Based on Enhanced Recovery after Surgery Concept to Elderly Patients Undergoing Total Knee Replacement” [1] due to concerns that the peer-review process has been compromised.

Following an investigation conducted by the Hindawi Research Integrity team [2], significant concerns were identified with the peer reviewers assigned to this article; the investigation has concluded that the peer-review process was compromised. We therefore can no longer trust the peer-review process, and the article is being retracted with the agreement of the Chief Editor.

## References

[1] Zhang J., Che J., Sun X., Ren W. (2022). Clinical Application of Perioperative Anaesthesia Management Based on Enhanced Recovery after Surgery Concept to Elderly Patients Undergoing Total Knee Replacement. *Computational Intelligence and Neuroscience*.

[2] Ferguson L. (2022). Advancing Research Integrity Collaboratively and with Vigour. https://www.hindawi.com/post/advancing-research-integrity-collaboratively-and-vigour/.

